# Nanostructured, Metal-Free Electrodes for the Oxygen Reduction Reaction Containing Nitrogen-Doped Carbon Quantum Dots and a Hydroxide Ion-Conducting Ionomer

**DOI:** 10.3390/molecules27061832

**Published:** 2022-03-11

**Authors:** Ashwini Reddy Nallayagari, Emanuela Sgreccia, Maria Luisa Di Vona, Luca Pasquini, Florence Vacandio, Philippe Knauth

**Affiliations:** 1 Aix Marseille University, CNRS, MADIREL (UMR 7246) and International Laboratory: Ionomer Materials for Energy, Campus St Jérôme, 13013 Marseille, France; nallayagari.ashwinireddy@students.uniroma2.eu (A.R.N.); luca.pasquini@univ-amu.fr (L.P.); florence.vacandio@univ-amu.fr (F.V.); 2 University of Rome Tor Vergata, Department Industrial Engineering and International Laboratory: Ionomer Materials for Energy, 00133 Roma, Italy; emanuela.sgreccia@uniroma2.it

**Keywords:** electrocatalysis, carbon materials, Koutecky–Levich plots, Tafel plots, capacitance

## Abstract

In this work, we studied the combination of nitrogen-doped carbon quantum dots (N-CQD), a hydroxide-ion conducting ionomer based on polysulfone (PSU) and polyaniline (PANI), to explore the complementary properties of these materials in high-performance nanostructured electrodes for the oxygen reduction reaction (ORR) in alkaline solution. N-CQD were made by hydrothermal synthesis from glucosamine hydrochloride (GAH) or glucosamine hydrochloride and N-Octylamine (GAH-Oct), and PSU were quaternized with trimethylamine (PSU-TMA). The nanocomposite electrodes were prepared on carbon paper by drop-casting. Furthermore, we succeeded in preparing PSU-TMA + PANI + GAH-Oct fibers by electrospinning. The capacitance of the electrodes was investigated by cyclic voltammetry and impedance spectroscopy, which gave similar trends. The ORR was investigated by linear sweep voltammetry at rotating disk electrode speeds between 250 and 2000 rpm in an oxygen-saturated 1 M KOH solution. Koutecky–Levich plots showed that four electrons were exchanged for nanocomposite electrodes containing CQD. The highest reduction currents were measured for the electrodes containing GAH-Oct. The Tafel plots gave the lowest slope and the most positive half-wave potential for PSU-TMA + PANI + GAH-Oct fibers. The best electrocatalytic activity of this electrode could be related to the high amount of graphitic nitrogen in GAH-Oct. Long-term cycling tests showed no significant modification of the onset potential, but a change of the current in the mass transport limited region, indicated the evolution of the microstructure of the nanocomposite ORR electrode modifying the mass transport conditions during the first 400 cycles before reaching stationary conditions. FTIR spectra were used to study possible electrode degradation after the ORR in 1 M KOH: the only change was due to the reaction of PANI emeraldine salt to emeraldine base, whereas the other constituents of the multiphase electrode did not show any degradation.

## 1. Introduction

The oxygen reduction reaction (ORR) is of particular theoretical and practical interest, for example in the respiratory reduction of O_2_ in cell metabolism [1] or the cathode of fuel cells [2,3,4] or other electrochemical devices [5]. It is well-known that the ORR is particularly difficult in acidic conditions, where Pt or other noble metals are needed as electrocatalysts if a four-electron reduction is desired. Less expensive electrocatalysts, such as polyaniline (PANI), might be used in acidic media if a two-electron reduction pathway to hydrogen peroxide that generates less power is acceptable [6,7,8]. The formation of hydrogen peroxide can however degrade the PEMFC components. In basic conditions, the ORR has a faster kinetic, and the four-electron reduction was demonstrated as feasible on various carbon-based electrode materials without the addition of noble metals [9,10,11,12,13,14]:

(1) $$O_{2}+4\;e^{-}+2\;H_{2}O\;\to 4\;OH^{-}\;\;\;\;\;\;\;\;\;\;\;E_{1}^{{}^{\circ}}=0.401\;V$$

Substitutional N-doping is a powerful method to improve graphitic carbon materials for various applications, including the catalysis of important chemical reactions, such as the ORR [15,16,17,18]. N-doped carbon quantum dots (CQD) can catalyze the electrochemical ORR in alkaline conditions with a size-dependent activity [15]. The two-electron peroxide pathway is preferred in acidic conditions, whereas the four-electron mechanism predominates in alkaline electrolytes [19]. The ORR electrocatalytic activity and the H_2_O/H_2_O_2_ selectivity are further influenced by oxygen-containing moieties [20], such as quinone-like groups, which are capable of O_2_ adsorption and are efficient mediators of the two-electron reduction of O_2_ to HO_2_*^−^* and the subsequent reduction to HO^−^ [6,19]:

(2) $$O_{2}+2\;e^{-}+H_{2}O\;\to HO_{2}^{-}+OH^{-}\;\;\;\;\;\;\;\;\;\;\;E_{2}^{{}^{\circ}}=-0.065\;V$$

(3) $$HO_{2}^{-}+2\;e^{-}+H_{2}O\;\to 3\;OH^{-}\;\;\;\;\;\;\;\;\;\;\;\;\;\;\;\;\;\;\;\;E_{3}^{{}^{\circ}}=0.867\;V$$

We have recently reported the advanced synthesis of CQD with average sizes in the nanometer range by pyrolysis, microwave irradiation and hydrothermal treatment. The content and position of N in the CQD can be tailored by functionalization with various nitrogen-containing compounds and the synthesis method and conditions [21]. CQD prepared by hydrothermal synthesis from glucosamine hydrochloride (GAH) and from glucosamine hydrochloride with the addition of N-octylamine (GAH-Oct) contains a substantial amount of nitrogen in the CQD lattice, and the presence of passivating N-octyl surface groups in GAH-Oct can adjust the compatibility with other electrode components. GAH and GAH-Oct are explored in this work as electrocatalysts for the ORR in alkaline conditions. The CQD are deposited on carbon paper by drop-casting and mixed with an anion exchange polymer, poly(sulfone trimethylammonium chloride) (PSU-TMA) to optimize the transport of OH^−^ ions near the catalytically active centers. Polyaniline (PANI), that reported to present interesting catalytic properties for the ORR, was added in some electrodes to verify if it boosts the catalytic performance [22,23,24]. The combination of these materials with complementary properties has never previously been investigated and is expected to provide high-performance nanostructured electrodes for the ORR.

## 2. Results

### 2.1. X-ray Diffraction

**Figure 1:**
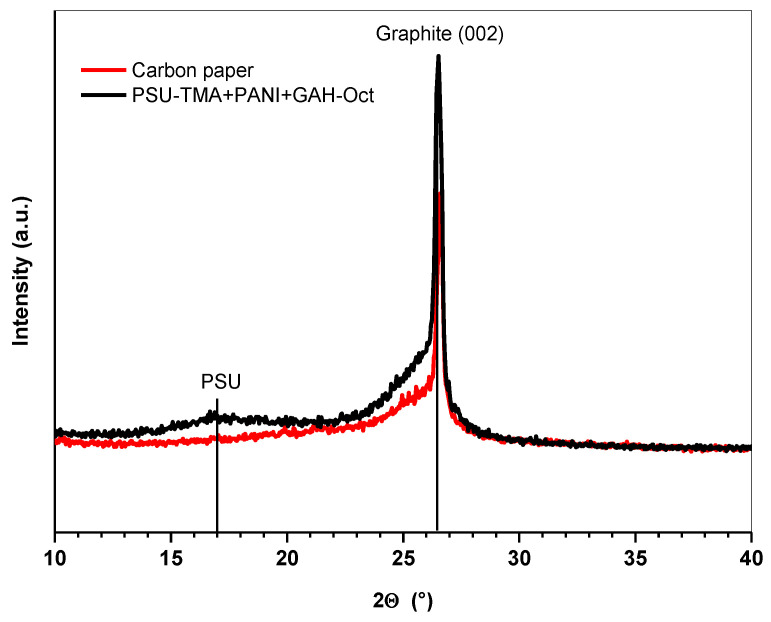
XRD patterns of carbon paper and PSU-TMA + PANI + GAH-Oct electrode.

The X-ray diffractogram of a PSU-TMA + PANI + GAH-Oct multiphase electrode on carbon paper is shown in Figure 1. A sharp peak can be observed at 26.5° corresponding to the (002) plane of hexagonal graphite. The shoulder centered at a slightly lower Bragg angle might be attributed to N-doped CQDs [25]. The broad signal around 17° can be assigned to amorphous PSU-TA because the main peak of polysulfone is located at this 2Θ value [26]. The main peak of PANI at 26° is hidden by the graphite peak.

### 2.2. Electrode Capacitance Measurements

#### 2.2.1. CV

**Figure 2:**
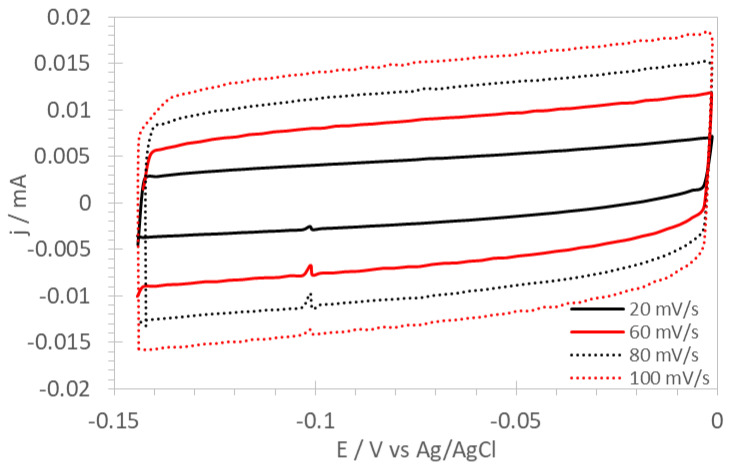
Cyclic voltammograms of PSU-TMA + PANI + GAH-Oct fibers.

Figure 2 shows cyclic voltammograms for PSU-TMA + PANI + GAH-Oct fibers at scan rates between 20 and 100 mV/s in the potential range between 0 and −0.15 V vs. Ag/AgCl, where no Faradaic reactions take place. the expected approximately rectangular shape of the curves can be observed. Similar curves for the other electrodes are reported in the Supplementary Materials.

The double-layer capacitance C is determined from the non-Faradaic current density at various scan rates, according to Equation (4):

(4) $$j=C\cdot \frac{dU}{dt}$$

**Figure 3:**
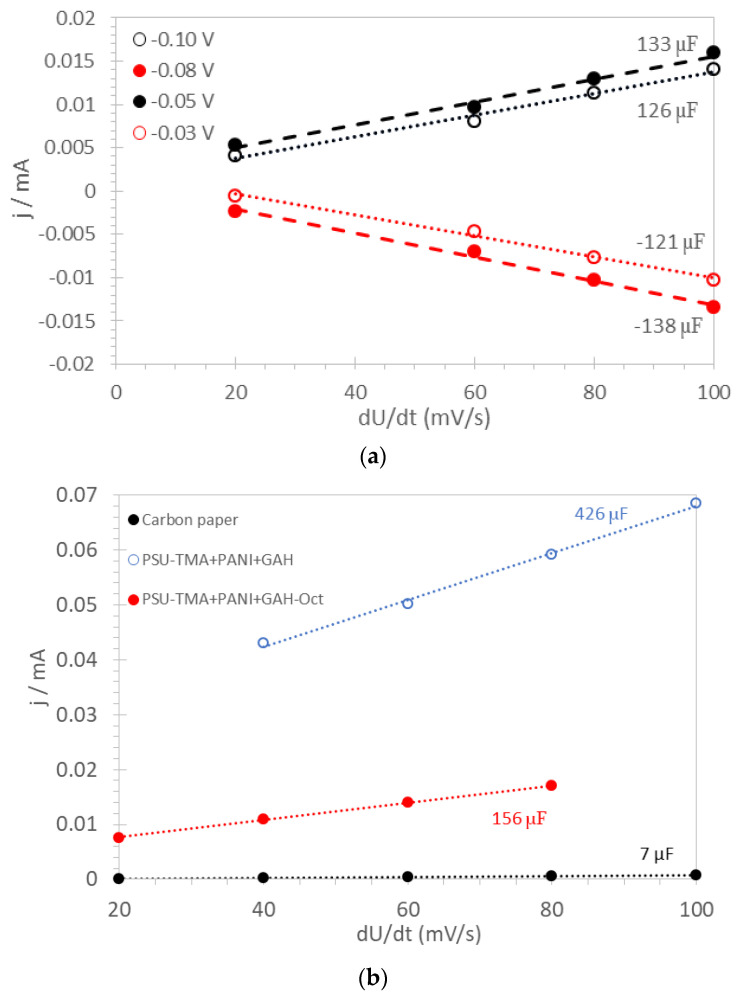
Capacitance determination for (**a**) PSU-TMA + PANI + GAH-Oct fiber electrode; (**b**) other electrodes.

Typical linear plots of the current density *j* vs. the scan rate *dU/dt* are presented in Figure 3a for the PSU-TMA + PANI + GAH-Oct fiber electrode. The average capacitance value *C*, determined from the slope of the straight lines according to Equation (4), is 130 μF and the consistency of the values is good. Plots for other electrodes are shown in Figure 3b; the average double-layer capacitance values are reported in Table 1.

The capacitance of carbon paper is much lower than that of any nanocomposite electrode, which shows high electrochemically active surface areas (EASA). It may be noted that the fiber electrode does not have the highest EASA, probably because some agglomeration takes place during the preparation (see experimental). Drop-casting is a valid method to provide high surface area electrodes.

Dividing the capacitance by the geometrical electrode area gives values between 1.8 and 6.1 mF/cm^2^. Assuming a double layer capacitance value of 20 μF/cm^2^ for flat electrodes [27], the EASA would be between approximately 6 and 22 cm^2^. However, lower double-layer capacitance values were reported for carbon-based materials [28].

#### 2.2.2. Impedance Spectroscopy

**Figure 4:**
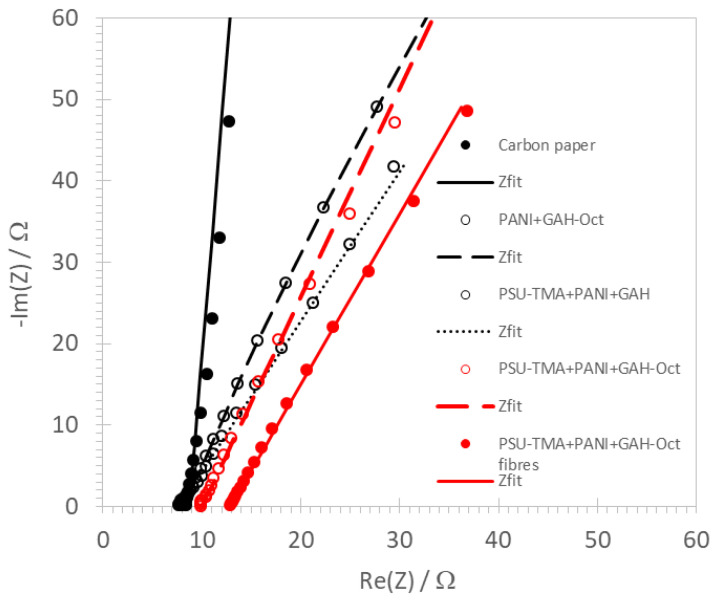
Impedance spectra of ORR electrodes at 1000 rpm in O_2_-saturated 1 M KOH.

Typical impedance spectra of various ORR electrodes are shown in Figure 4.

They were fitted using an equivalent circuit consisting of a series alignment of a resistance R (attributed to the electrolyte and the electrode) and a constant phase element (CPE) Q. The impedance of the CPE can be written:

(5) $$Z\left(Q\right)=\left(\frac{1}{Y}\right)\;{\left(i\omega \right)}^{-m\;\;}$$

where *i* is the imaginary unit, *ω* the angular frequency, *Y* the CPE value, and *m* the CPE exponent, which is characteristic of the physical nature of the element: a value near 1 indicates an imperfect capacitance [29]. The best-fit values of *R*, *Y*, and *m* are reported in Table 1. The resistance is small in all cases with little variations. For the carbon paper electrode, which presents a nearly ideal capacitance (*m* = 0.95), the *Y* value is consistent with the double layer capacitance determined by CV measurements (Table 1). The *Y* data for the other electrodes, if attributed to double-layer capacitances, are consistent with the trend from CV measurements showing that the electrode surface is much higher and more irregular, leading to a capacitance distribution and a lower CPE exponent [30]. The two highest values of *Y* correspond to the two lowest values of *m*, consistent with the high porosity of these drop-casted electrodes.

### 2.3. Cyclovoltammetry and Linear Sweep Voltammetry of ORR

**Figure 5:**
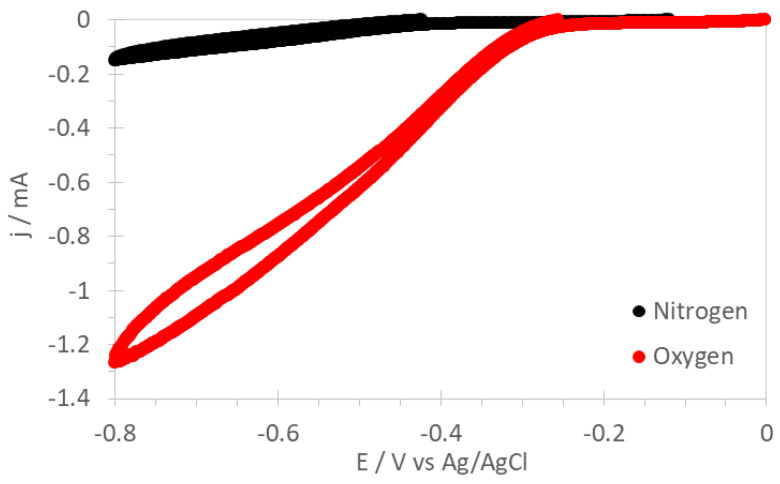
Cyclovoltammograms of PSU-TMA + PANI + GAH-Oct in N_2_- and O_2_-saturated 1 M KOH solution at 1500 rpm.

Figure 5 shows cyclovoltammograms of PSU-TMA + PANI + GAH-Oct in N_2_- and O_2_-saturated 1 M KOH solution. A well-defined reduction peak is observed with an onset potential around −0.2 V vs. Ag/AgCl in the O_2_-saturated, but not in the N_2_-saturated solution, showing that O_2_ is reduced on the electrode with PSU-TMA + PANI + GAH-Oct.

**Figure 6:**
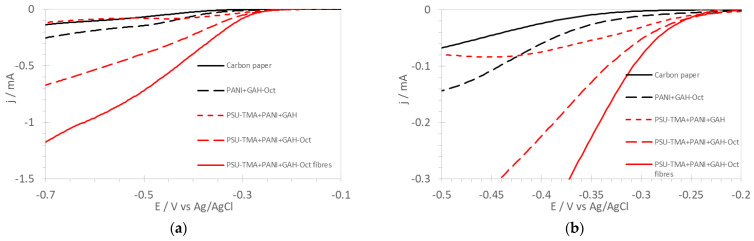
(**a**) Linear sweep voltammograms of the ORR in O_2_-saturated 1 M KOH with RDE at 1500 rpm for various electrodes. (**b**) Focus on the low current region.

Figure 6 presents linear sweep voltammograms on RDE at 1500 rpm for carbon paper and nanocomposite electrodes. The LSV for an electrode without PSU-TMA is also shown for comparison. The onset potentials determined from these curves are reported in Table 1. Literature data for the ORR performance of various nitrogen-doped nanocarbon catalysts show onset potentials between −0.08 and −0.32 V vs. Ag/AgCl [31]. The large catalytic activity of PSU-TMA + PANI + GAH-Oct illustrates the importance of nitrogen doping of CQD for the ORR electrocatalytic activity because this sample contains a much larger amount of nitrogen in the carbon lattice. Focusing on the low current region (Figure 6b) allows observation of further interesting details of the curves for ORR electrodes containing CQD. Of note, for example, is that the behavior of PSU-TMA + PANI + GAH is also quite promising at low overpotential, showing some ability as an electrocatalyst, but at higher overvoltage, mass-transport limitations are observed. The CQD passivation by N-octyl groups reduces the aggregation and favors the homogeneity of the nanocomposites containing GAH-Oct. The impossibility of obtaining electrospun fibers with GAH is probably due to the same reason. Somewhat similar is the curve for PANI + GAH-Oct showing significant mass-transport limitations. Here the absence of PSU-TMA is probably detrimental for the OH^−^ transport.

**Figure 7:**
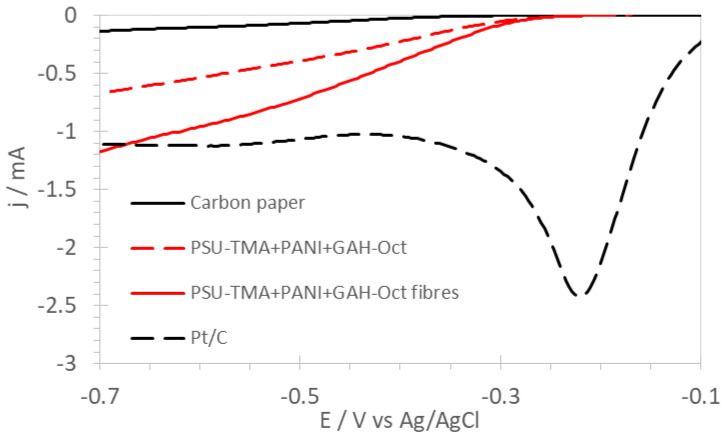
Linear sweep voltammograms of nanostructured electrodes of the ORR in O_2_-saturated 1 M KOH with RDE at 1500 rpm in comparison with commercial Pt/C foil.

A comparison of LSV of PSU-TMA + PANI + GAH-Oct electrodes with commercial Pt/C foil is shown in Figure 7.

**Figure 8:**
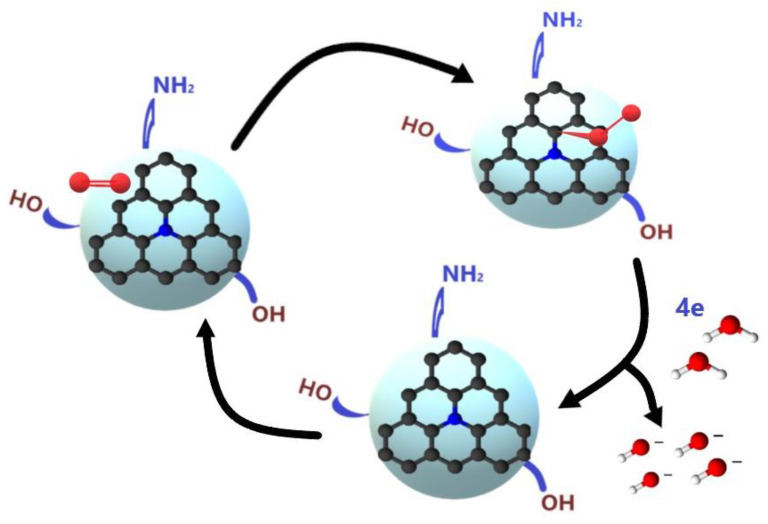
Schematic representation of the ORR on carbon quantum dots.

The electrocatalytic activity of N-doped carbon materials is attributed to the capacity of the nitrogen to induce polarization on the neighboring carbon, the active center for the chemisorption of O_2_ as indicated in Figure 8 [32].

The presented results show that ORR electrodes containing GAH-Oct have a higher catalytic activity than that with GAH, although the latter electrode has a higher electrochemically surface area. A convincing interpretation can be based on the position of nitrogen atoms inside the CQD and their influence on the ORR electrocatalytic activity. It was demonstrated previously that graphitic nitrogen has a distinctly higher catalytic activity than pyridinic or pyrrolic nitrogen [19]. We have shown by XPS analysis that GAH-Oct contains the greatest amount of nitrogen and indeed essentially in graphitic position (Pyrrolic/Amine/Amide 1 at %, Graphitic 7.4 at %, Pyridinic oxide 1.6 at % [21]), whereas GAH contains mostly pyridinic and pyrrolic and less graphitic nitrogen (Pyridinic 2.5 at %, Pyrrolic/Amine/Amide 3.7 at %, Graphitic 1.6 at % [21]). Furthermore, the presence of oxygen-containing functionalities, especially quinone moieties [19], carbonyl (C=O), ester, and carboxyl groups [20], is critical for the enhanced electrocatalytic activity vs. the ORR. 

**Figure 9:**
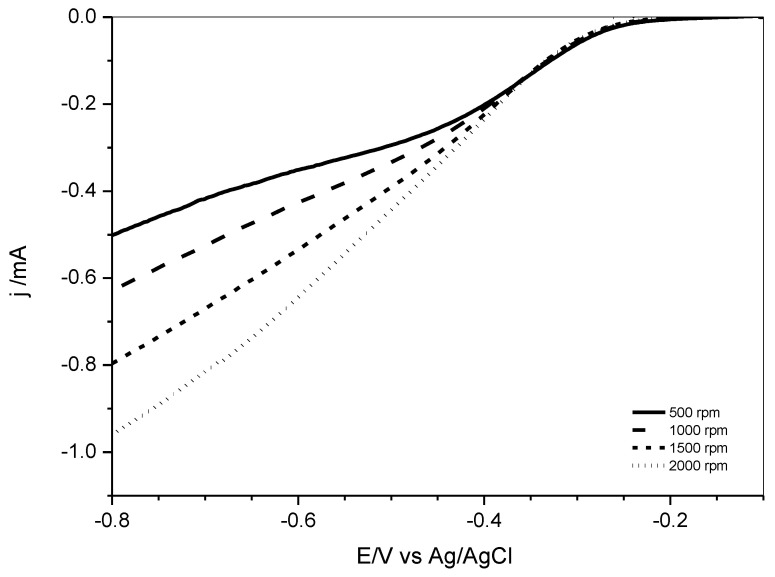
Linear scan voltammograms in O_2_-saturated 1 M KOH solution with various rotating speeds for PSU-TMA + PANI + GAH-Oct.

Linear scan voltammograms (LSV) at various rotational speeds of the RDE are shown in Figure 9 for PSU-TMA + PANI + GAH-Oct. The LSV for the other nanocomposite electrodes is reported in the Supplementary Materials.

The number *n* of transferred electrons per oxygen molecule can be determined using the Koutecky–Levich equation between the current *j* and the rate of the rotating-disk electrode *ω* [33,34]:

(6) $$\frac{1}{j}=\frac{1}{j^{k}}+\frac{1}{B\cdot \omega^{1/2}}$$

In this equation, *j^k^* is the kinetic current and *B* is the Levich slope, which is given by the relation:

(7) $$B=0.62\cdot A\cdot n\cdot F\cdot c\left(O_{2}\right)\cdot D{\left(O_{2}\right)}^{\frac{2}{3}}\cdot v^{-\frac{1}{6}}$$

**Figure 10:**
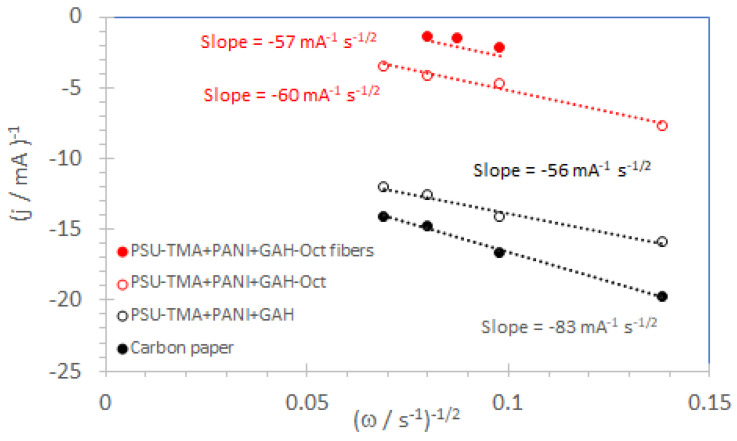
Koutecky–Levich plots were obtained from the LSV curves in Figure 9 and in the Supplementary Materials at −0.5 V vs. Ag/AgCl reference electrode.

Here, *A* is the geometrical electrode area, *F* the Faraday constant (96,485 C mol^−1^), *c*(O_2_) the saturation concentration of oxygen in 1 M KOH (8 × 10^−7^ mol cm^−3^ [35,36]), *D*(O_2_) the oxygen diffusion coefficient (1.5 × 10^−5^ cm^2^ s^−1^ in 1 M KOH [35]), and *ν* the kinetic viscosity of the 1 M KOH solution (0.0094 cm^2^ s^−1^) [37]. Typical Koutecky–Levich plots are shown in Figure 10.

The slopes of Koutecky–Levich plots for nanocomposite electrodes containing GAH and GAH-Oct are in good agreement with the value for 4 transferred electrons per oxygen molecule (56 mA^−1^ s^−1/2^). The number of exchanged electrons is reported in Table 2. It can be concluded that CQD catalyze four-electron reduction, as previously reported for other carbonaceous ORR catalysts in basic solutions [20]. For carbon paper, *n* < 3, so that more hydrogen peroxide than water is formed by the ORR.

### 2.4. Tafel Plots

**Figure 11:**
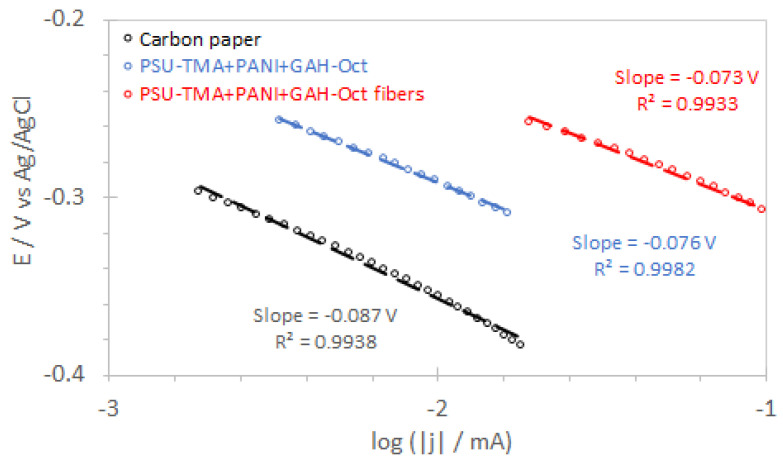
Tafel plots were obtained from LSV curves at 1500 rpm.

Tafel plots for carbon paper and electrodes with GAH-Oct are shown in Figure 11. The Tafel slopes can be obtained according to Equation (8) relating the absolute value of the cathodic overpotential |*η*| and the current density |*j*| [38]:

(8) $$\left|\eta \right|=\frac{2.3\cdot RT}{\alpha nF}\log\left(\frac{\left|j\right|}{j{}^{\circ}}\right)$$

In this equation, *R* is the ideal gas constant (*R* = 8.314 J K^−1^ mol^−1^), *T* the absolute temperature, *α* the symmetry coefficient, assumed to be 0.5, and *j*° the exchange current density. The slope for carbon paper is in the order of 90 mV/decade, intermediate between values reported in the literature for low current densities (2.3*·RT*/*F*) indicating a pseudo-two-electron-process to be the rate-determining step [12,36], and high current densities (2.3·*RT*/0.5·*F*) [6,38], where the first electron transfer on chemisorbed oxygen is assumed to be rate-determining [19,39].

The lower Tafel slopes with GAH-Oct indicate a better catalytic activity. The lowest value is observed for PSU-TMA + PANI + GAH-Oct fibers, which present also the most positive half-wave potential (Table 2), confirming the best electrocatalytic properties of this nanocomposite electrode. Half-wave potentials reported in the literature for various nitrogen-doped nanocarbon catalysts are between −0.18 and −0.38 V vs. Ag/AgCl [31].

### 2.5. Stability Tests

**Figure 12:**
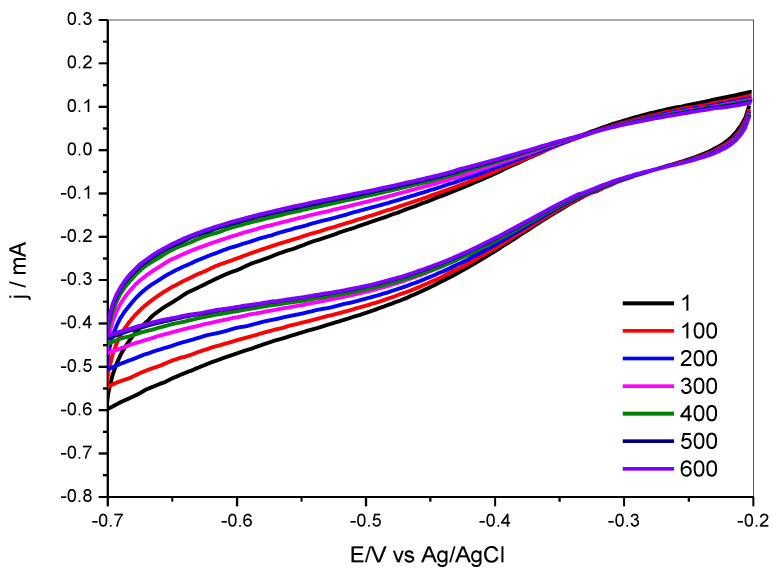
Stability test: cyclovoltammograms for PSU-TMA + PANI + GAH-Oct in O_2_ saturated 1 M KOH as a function of the cycle number.

A cycling test for the PSU-TMA + PANI + GAH-Oct electrode is shown in Figure 12. Cyclovoltammograms over 600 cycles show that the onset potential does not change significantly, indicating that the electrocatalytic properties of the electrodes are preserved. The current decreases, however, in the mass transport-limited region around −0.5 V during the first 400 cycles, indicating a microstructural modification of the electrode, which modifies the charge transport, before stabilization after about 400 cycles, where stationary conditions are reached.

### 2.6. FTIR Spectroscopy

**Figure 13:**
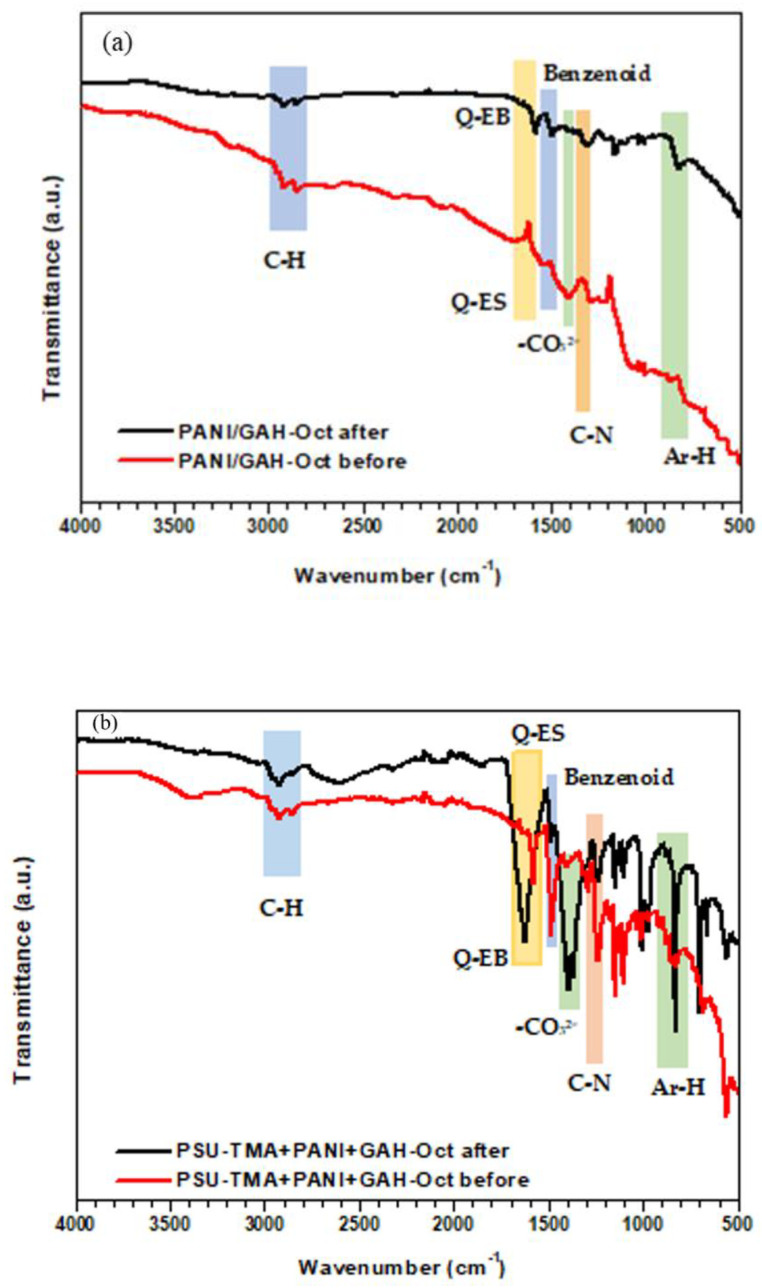
FTIR spectra of nanocomposite ORR electrodes before and after use (Q: quinoid, ES: emeraldine salt, EB: emeraldine base). (**a**) PANI + GAH-Oct; (**b**) PSU-TMA + PANI + GAH-Oct.

FTIR spectra of PANI-GAH-Oct and PSU-TMA + PANI + GAH-Oct, recorded before and after the electrocatalytic experiments, are presented in Figure 13 in order to analyze the stability of the ORR electrodes in alkaline solution.

**Scheme 1:**
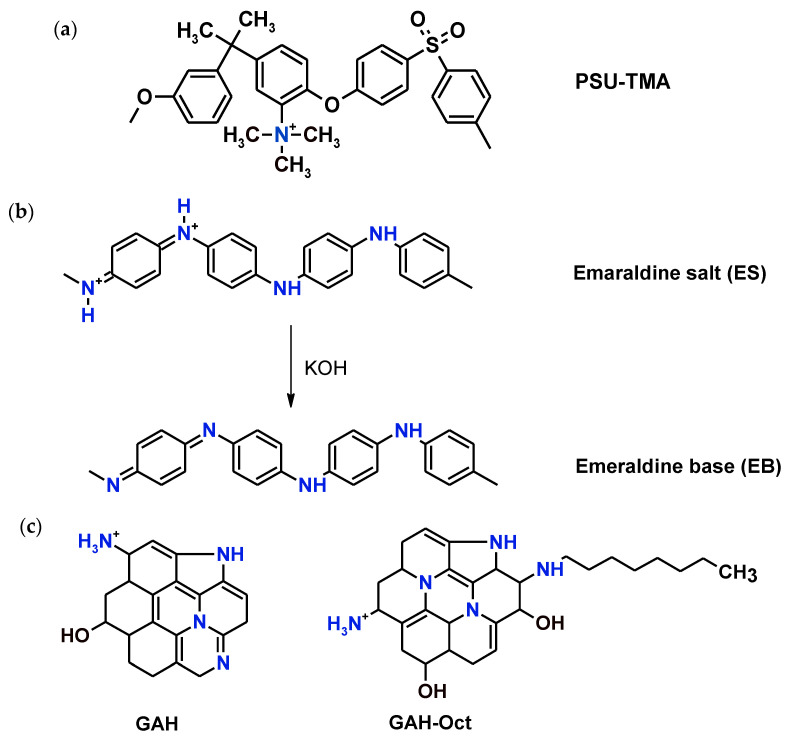
Chemical formulas of (**a**) PSU-TMA, (**b**) PANI (emeraldine salt and base) and (**c**) simplified structures of CQD.

PANI-ES is neutralized to PANI-EB after immersion in the alkaline solution used for the ORR (Scheme 1b). The formation of the emeraldine base is evident in the FTIR analysis. Figure 13a shows the FTIR spectra of the electrodes formed by PANI + GAH-Oct before and after the measurements in alkaline condition. The adsorptions in the range 2820–3000 cm^−1^ originate from the C-H stretch of octyl groups attached to CQD. The main difference in the spectra is the shift to higher wavenumber of the peak due to the stretching vibration of the nitrogen quinoid ring, which is responsible for the catalytic activity of PANI [22,23,24], present at 1600 cm^−1^ for EB, and 1565 cm^−1^ for ES [40,41]. The benzenoid portion of PANI is present at 1500 cm^−1^ in EB, and only as a shoulder at 1480 cm^−1^ in ES. The broad peak at 1400 cm^−1^, which is observed only in the initial sample, can be attributed to the asymmetric stretching of carbonate groups, the counter-ion of ammonium groups initially present in GAH-Oct. The peak disappears after treatment in basic solution [42]. The C-N stretching absorption for aromatic amines is around 1307 cm^−1^ for EB, and 1298 cm^−1^ for ES. From the out-of-plane C-H bonding region, it is possible to estimate the substitution pattern of the aromatic rings, resulting in a peak at 820 and 800 cm^−1^ for EB and ES respectively, corresponding to 1,4-substitution. The same behavior is also observed in the spectra of GAH-Oct alone, presented in the Supplementary Materials together with the spectra of carbon paper and PSU-TMA + PANI + GAH.

More complex are the spectra of PSU-TMA + PANI + GAH-Oct electrodes (Figure 13b), because of the presence of the PSU-TMA component. The anion exchange polymer remains unchanged during the ORR. The spectra show in the range 3000–2850 cm^−1^ the C-H stretching due to the overlapping vibrations of methyl groups in polysulfone and trimethylammonium, and the aliphatic chain of GAH-Oct. At 1250 cm^−1^ the vibration of the ether link of PSU is visible [43]. As observed before, the main differences are due to the equilibria between ES and EB: the peak due to the stretching vibration of the quinoid ring, in EB at 1610 cm^−1^ is shifted to 1580 cm^−1^ in ES. An intense peak appears after the ORR at 1380–1400 cm^−1^, attributed to carbonatation of PSU-TMA (the initial ionomer is in chloride form). In conclusion, the constituents of the nanocomposite electrode do not degrade during the ORR.

## 3. Experimental

### 3.1. Materials

Polysulfone was purchased from Solvay (PSU, MW = 55,500 g/mol). Polyaniline (emeraldine salt, PANI-ES, MW > 15,000 g/mol), D-(+)-glucosamine hydrochloride (GA), N-octylamine (Oct), N,N, N-trimethylamine (TMA, 4.2 M in EtOH), N-methylpyrrolidone (NMP), dimethylformamide (DMF) and the other chemicals were reagent-grade and were used as received from Sigma-Aldrich. Carbon paper (AvCarb EP55) and Pt/C foil were obtained from Fuel Cell Store.

### 3.2. Synthesis

- *GAH and GAH-Oct*

The synthesis of GAH and GAH-Oct by a hydrothermal treatment was previously presented [21]. In brief, 0.45 g of glucosamine hydrochloride (2.1 mmol) was dissolved in 1 mL H_2_O or in 1 mL H_2_O and 0.4 mL N-octylamine (2.1 mmol) for GAH and GAH-Oct, respectively. The mixtures were stirred for 40–50 min at 50 °C to obtain uniform solutions, then transferred in an autoclave and inserted into an oven at 190 °C for 12 h. The autoclave was then cooled down to room temperature and the solid was dispersed in deionized water for GAH and chloroform for GAH-Oct, which is hydrophobic. The final solutions were centrifugated. The solids were used for the electrode preparation. The characterization of GAH and GAH-Oct and transmission electron microscopy images are reported in [21].

*PSU-TMA*

The anion exchange ionomer was prepared by the chloromethylation route followed by amination as described in [44]. Chloromethylated PSU (PSU-CM) was obtained from paraformaldehyde, trimethylchlorosilane, and SnCl_4_ in chloroform. After purification, the degree of chloromethylation was 1.2. PSU-TMA was obtained by reacting PSU-CM with TMA in DMSO. The resulting degree of amination was 0.9 and the ion exchange capacity (IEC) was 1.8 meq/g.

The chemical structures of PSU-TMA, PANI (emeraldine salt and base), and CQD, are represented in Scheme 1.

### 3.3. Electrode Fabrication

The electrodes were made by coating carbon paper with 5 μL of a suspension of the constituents using a microsyringe. The coated electrodes were dried at 80 °C for 24 h. The preparation of the suspensions is described below.

*PANI + GAH-Oct*

An amount of 0.15 g of finely powdered GAH-Oct was dispersed in 0.60 g of DMF under vigorous stirring. Later, 0.07 g of PANI was added to the above solution.

*PSU-TMA + PANI + GAH*

An amount of 0.45 g of PSU-TMA in 2.0 g of DMSO was inserted into a small, closed glass vial with 0.07 g of PANI, and 0.15 g of finely powdered GAH was added with 0.60 g of DMF as solvent.

*PSU-TMA + PANI + GAH-Oct*

The same concentration of PSU-TMA and PANI in DMSO was maintained and 0.15 g GAH-Oct with 0.68 g NMP was added to disperse the materials well. 

In all cases, the final solutions were kept overnight under stirring before drop-casting the electrodes.

Furthermore, fibers of PSU-TMA + PANI + GAH-Oct were obtained by electrospinning using a Starter Kit-Random (Linari Engineering). A 10 mL syringe with a 0.8 mm diameter needle was mounted on the syringe pump setting a flow rate of 0.04 mL/h. The fibers were electrospun at an applied voltage of 15 kV with a needle/collector (carbon paper) distance of 16 cm. 

### 3.4. Electrode Characterization

**Figure 14:**
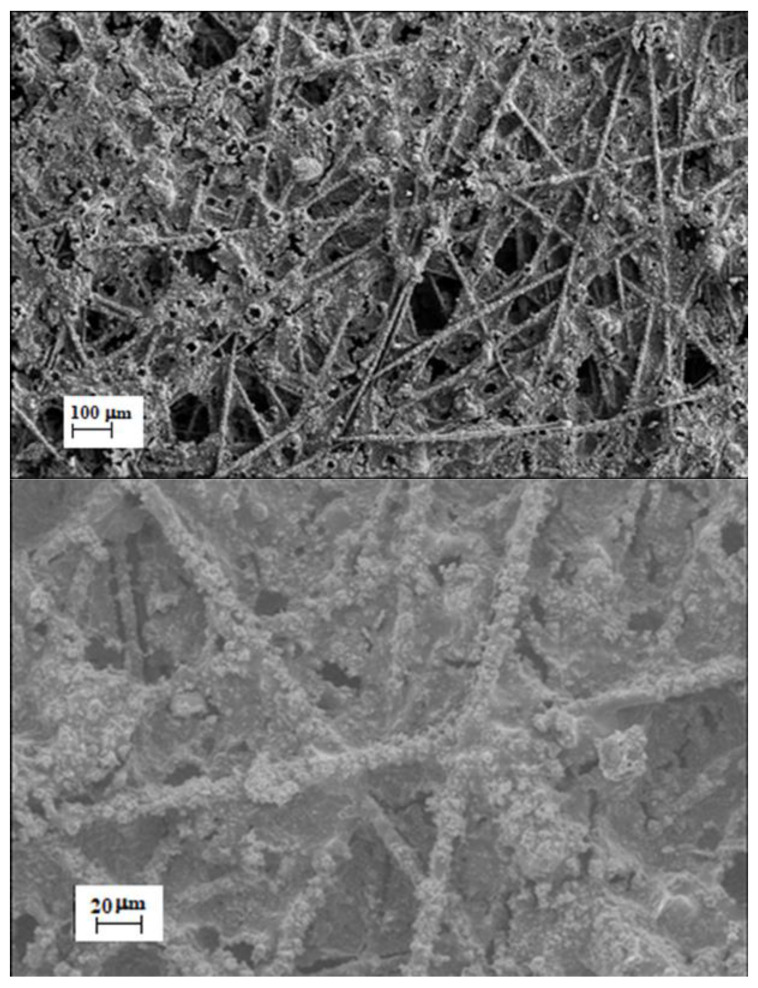
SEM micrographs with a different magnification of PSU-TMA + PANI + GAH-Oct fibers.

SEM micrographs (ZEISS Gemini SEM 500, acceleration voltage 5 kV) of a typical electrospun electrode containing PSU-TMA, PANI and GAH-Oct are shown in Figure 14 with two different magnifications. The three phases present in the fibrous electrode are not miscible, so they can be observed separately: the microfibres are PSU-TMA, whereas PANI and GAH-Oct are agglomerated on top. 

Despite much effort, similar attempts to prepare electrospun fibers with GAH or without PANI were, unfortunately, unsuccessful.

X-ray diffractograms were recorded using a Bruker D5000 diffractometer with Cu Kα radiation (λ = 0.15406 nm, 38 kV, 30 mA), 0.04° steps, and 2 s step time. 

FTIR spectra were recorded using a Perkin Elmer Spectrum Two spectrometer (Waltham, Massachusetts, United States) equipped with an ATR crystal diamond module in a wavenumber range of 500–4000 cm^−1^ with a resolution of 0.5 cm^−1^.

### 3.5. Electrochemical Measurements

Cyclic voltammetry (CV), linear sweep voltammetry (LSV), and electrochemical impedance spectroscopy (EIS) measurements were performed using a Biologic VMP3 potentiostat with a three-electrode cell. 

A glassy carbon rotating disk electrode with 0.07 cm^2^ area (RDE, OrigaTrod, OriaLys) was used as the working electrode. The coated carbon paper samples were fixed on the glassy carbon disk. A 4 cm^2^ platinum foil was the counter-electrode and saturated Ag/AgCl served as reference electrode (E = 0.197 V vs. NHE). All experiments were conducted at room temperature. The O_2_- and N_2_-saturated 1 M aqueous KOH solutions were obtained by bubbling the pure gases for at least 40 min through the liquid. 

The scan rate for the CV and the LSV measurements was 20 mV/s with various rotating speeds of the RDE between 250 and 2000 rpm. The long-term cycling tests were performed between −0.2 and −0.7 V vs. Ag/AgCl at 1500 rpm with a 50 mV/s scan rate. The impedance spectra were recorded using a Biologic VMP3 with an a.c. amplitude of 20 mV in a frequency range between 1 Hz and 1 MHz.

## 4. Conclusions

We studied the electrocatalytic properties for the ORR of nanocomposites combining a hydroxide ion-conducting ionomer (PSU-TMA), polyaniline (PANI), and CQD prepared by hydrothermal synthesis from glucosamine (GAH) without, and with, N-octylamine (GAH-Oct). 

Application of the Koutecky–Levich equation to the linear sweep voltammograms of the ORR showed a four-electron reduction for the catalytic electrodes containing CQD. The best electrocatalytic activity was found for PSU-TMA + PANI + GAH-Oct fibers. The lower Tafel slope and most positive half-wave potential of this nanocomposite electrode could be attributed to the high amount of graphitic nitrogen in the CQD that was previously shown to improve the electrocatalytic activity for the ORR.

Long-term cycling experiments showed good stability of the onset potential, but the current decrease in the plateau region during the first 400 cycles showed a microstructural evolution of the nanocomposite electrode that modified the mass transport. The FTIR investigation of the nanocomposite electrodes before and after the ORR in alkaline solution mainly showed the neutralization of PANI emeraldine salt, but no significant degradation of the constituents of the multiphase electrodes.

## Figures and Tables

**Table 1:** Electrochemical properties of ORR electrodes: double-layer capacitance, C; resistance, R; CPE value, Y; exponent, m; and onset potential, E_onset_ (see text).

Sample	C (μF)	R (Ω)	Y (μF s^n−1^)	m	E_onset_ vs. Ag/AgCl (mV)
Carbon paper	7	8.7	9	0.95	−208
PANI + GAH-Oct	-	8.1	370	0.79	−205
PSU-TMA + PANI + GAH	430	7.7	1210	0.68	−179
PSU-TMA + PANI + GAH-Oct	160	16.6	840	0.65	−171
PSU-TMA + PANI + GAH-Oct fibers	130	12.9	330	0.72	−182

**Table 2:** Electrochemical properties of ORR electrodes: half-wave potential E_1/2_, Tafel slope B, number of exchanged electrons n (see text) at E = −0.5 V vs. Ag/AgCl.

Sample	E_1/2_/mV	B/mV	*n*
Carbon paper	−410	87	2.7
PSU-TMA + PANI + GAH-Oct	−345	76	3.8
PSU-TMA + PANI + GAH-Oct Fibers	−340	73	4.0

## Data Availability

Data is contained within the article or Supplementary Materials.

## Supplementary Materials

The following supporting information can be downloaded at: https://www.mdpi.com/article/10.3390/molecules27061832/s1. The cyclovoltammograms in the non-Faradaic region are reported for various electrodes in Figures S1–S3, Figure S1: Carbon paper; Figure S2: PSU-TMA+PANI+GAH; Figure S3: PSU-TMA + PANI + GAH-Oct; Linear scan voltammograms (20 mV/s) are reported in Figures S4–S7 for various electrodes in O2-saturated 1 M KOH solution with various rotating rates, Figure S4: Carbon paper; Figure S5: PANI+GAH-Oct; Figure S6: PSU-TMA+PANI+GAH; Figure S7: PSU-TMA+PANI+GAH-Oct fibres; The FTIR spectra for various electrodes are reported in Figures S8–S10 before and after the ORR in alkaline solution, Figure S8: Carbon paper; Figure S9: GAH-Oct; Figure S10: PSU-TMA + PANI + GAH.

## Sample Availability

Samples of the compounds are not available from the authors.
