# Supplementary material for: Nanostructured, Metal-Free Electrodes for the Oxygen Reduction Reaction Containing Nitrogen-Doped Carbon Quantum Dots and a Hydroxide Ion-Conducting Ionomer
Source: Molecules. 2022 Mar 11;27(6):1832. doi: 10.3390/molecules27061832 (PMC8953787; doi:10.3390/molecules27061832)
Supplement: Supplementary file 1 [file molecules-27-01832-s001.zip › molecules-1615557-supplementary.pdf]

Supporting information

Nanostructured, metal-free electrodes for the oxygen reduction reaction containing nitrogen-doped carbon quantum dots and a hydroxide ion-conducting ionomer

A. R. Nallayagari [1,2], *E. Sgreccia* [2], *M. L. Di Vona* [*,2],
L. Pasquini [1], *F. Vacandio* [1], *P. Knauth* [*,1]

[1] Aix Marseille Univ, CNRS, MADIREL (UMR 7246) and International Laboratory: Ionomer Materials for Energy, Campus St Jérôme, 13013 Marseille, France

[2] University of Rome Tor Vergata, Dep. Industrial Engineering and International Laboratory: Ionomer Materials for Energy, 00133 Roma, Italy

Capacitance measurements

The cyclovoltammograms in the non-Faradaic region are reported for various electrodes in Figures S1-S3.

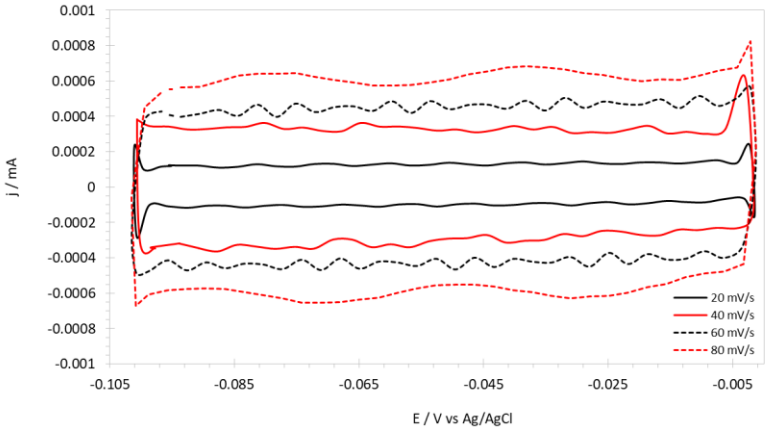

Figure S1. Carbon paper

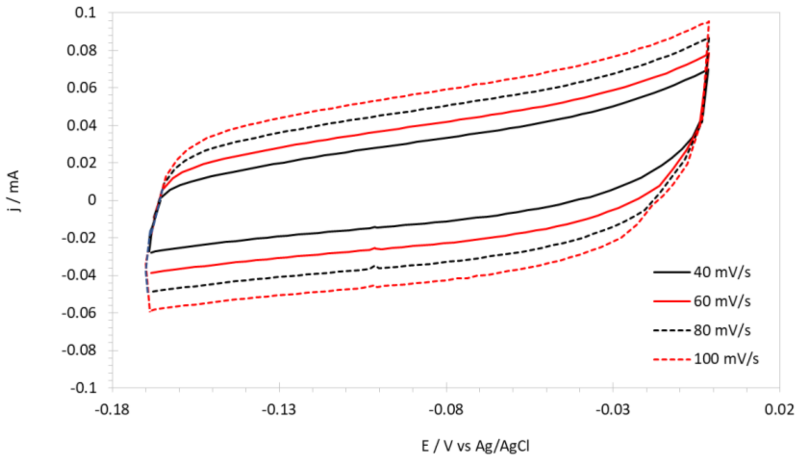

Figure S2. PSU-TMA+PANI+GAH

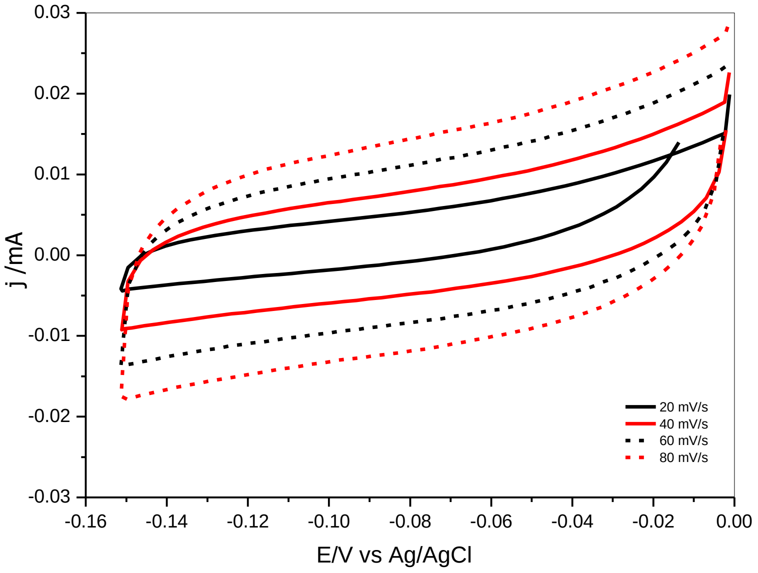

Figure S3. PSU-TMA+PANI+GAH-Oct

Linear sweep voltammetry

Linear scan voltammograms (20 mV/s) are reported in Fig. S4-S7 for various electrodes in O_2-saturated 1 M KOH solution with various rotating rates.

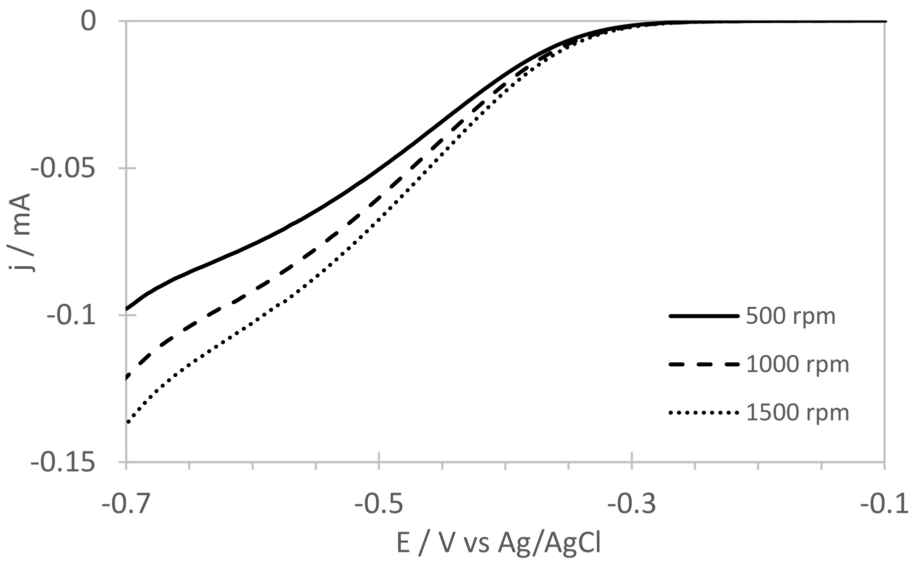

Figure S4. Carbon paper

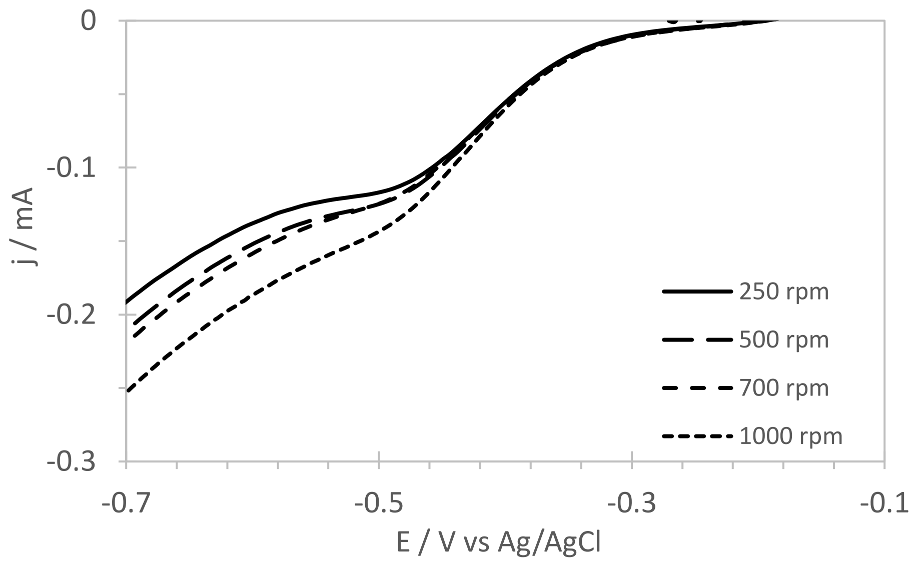

Figure S5. PANI+GAH-Oct

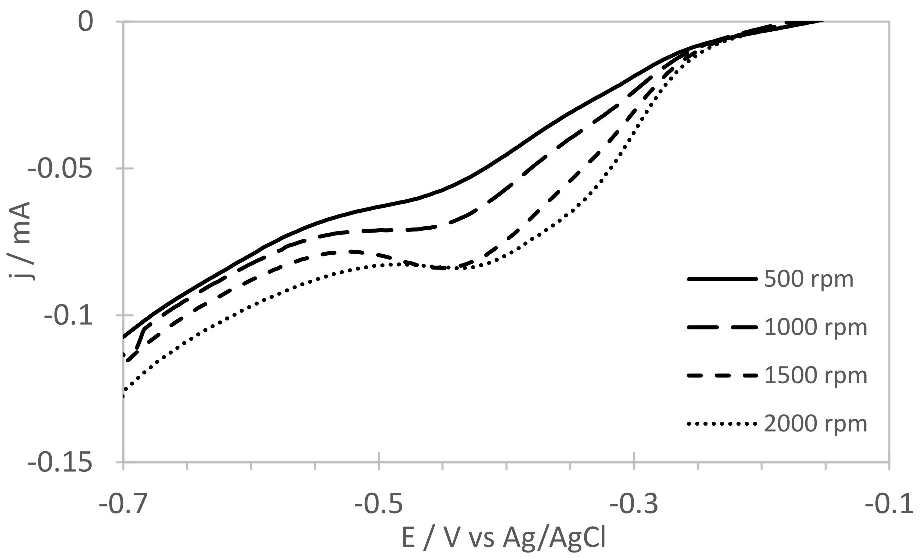

Figure S6. PSU-TMA+PANI+GAH

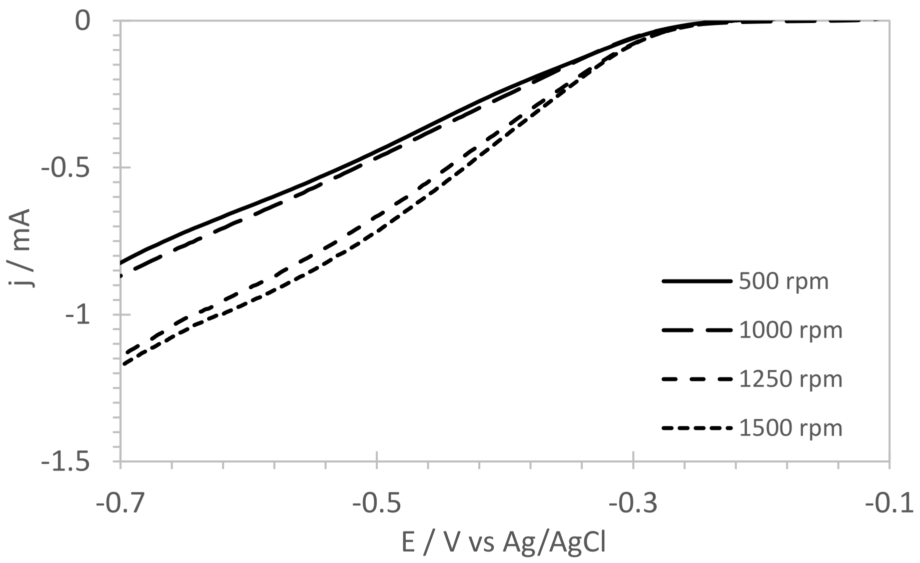

Figure S7. PSU-TMA+PANI+GAH-Oct fibres.

FTIR spectra

The FTIR spectra for various electrodes are reported in Fig. S8-S10 before and after the ORR in alkaline solution.

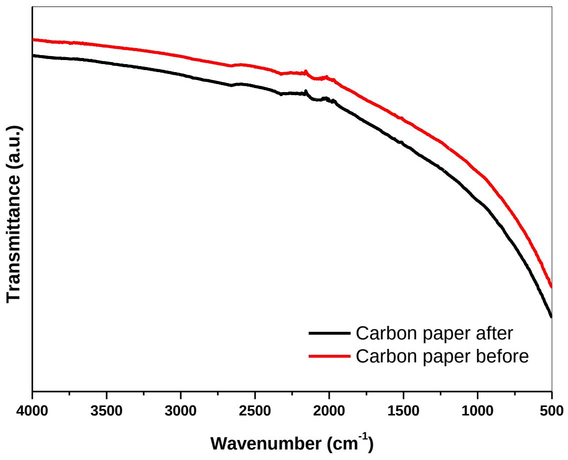

Figure S8. Carbon paper

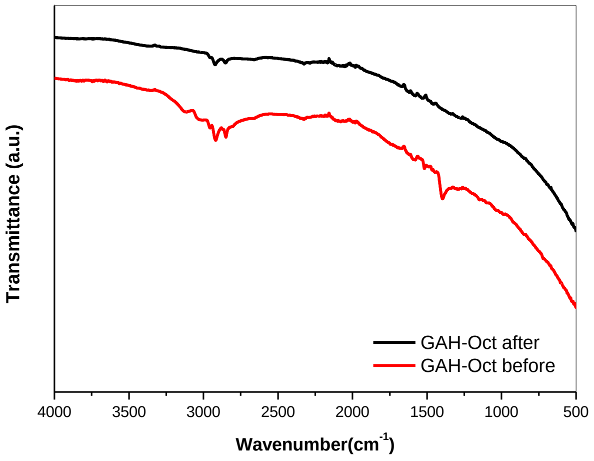

Figure S9. GAH-Oct

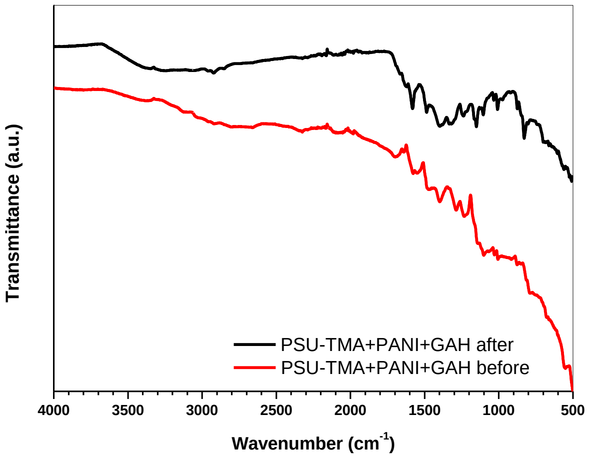

Figure S10. PSU-TMA+PANI+GAH
